# Efficacy of High-Dose Albendazole with Ivermectin for Treating Imported Loiasis, Italy

**DOI:** 10.3201/eid2508.190011

**Published:** 2019-08

**Authors:** Federico Gobbi, Dora Buonfrate, Francesca Tamarozzi, Monica Degani, Andrea Angheben, Zeno Bisoffi

**Affiliations:** IRCCS Sacro Cuore Don Calabria Hospital, Negrar di Valpolicella, Italy (F. Gobbi, D. Buonfrate, F. Tamarozzi, M. Degani, A. Angheben, Z. Bisoffi);; ISS (Istituto Superiore Sanità), Rome, Italy (F. Tamarozzi);; Università degli Studi di Verona, Verona, Italy (Z. Bisoffi)

**Keywords:** albendazole, DEC, diethylcarbamazine, efficacy, Italy, ivermectin, Loa loa, loiasis, meningitis/encephalitis, parasites, vector-borne infections

## Abstract

We describe the outcomes of 16 cases of imported loiasis in Italy. Patients had microfilaremia <20,000/mL and were treated with high-dose albendazole for 28 days and a single dose of ivermectin. This combination might be an effective treatment option in nonendemic areas, when diethylcarbamazine, the drug of choice, is not available.

*Loa loa* is a filarial nematode transmitted by tabanid flies of the genus *Chrysops*, which inhabits forested areas of West and Central Africa (*1*). It is estimated that >10 million people are infected with this parasite (*2*). Adult worms move under the skin or in the intermuscular fascia and can produce microfilariae. Loiasis can cause a wide range of symptoms, most frequently migrant edemas (Calabar swellings). Severe neurologic complications have also been reported (*2*). A retrospective study showed increased mortality rates in patients with high microfilaremia (*3*), indicating that this infection is not a benign condition, as previously thought. 

Three drugs are currently used to treat loiasis: diethylcarbamazine, ivermectin, and albendazole (*4*). Diethylcarbamazine is preferred but is usually unavailable outside of specific World Health Organization mass drug administration programs. In addition, multiple courses of diethylcarbamazine are often required to achieve a clinical and parasitological cure (*5*), and the drug should not be used in patients with high microfilaremia levels because of the risk of encephalopathy (*4*). Trials in loiasis-endemic countries showed that short courses of albendazole had little effect on *L. loa* infection (*6*,*7*), but longer treatments (200 mg 2×/d for 21 d) resulted in decreased microfilaremia, blood eosinophil levels, and antifilarial antibodies (*8*). In 2018, a patient with high microfilaremia was reported to be clear of infection after four 21-day courses of albendazole at a dose of 400 mg daily, followed by a single 150 µg/kg dose of ivermectin (*9*). We describe the outcomes of 16 cases of imported loiasis in Italy with high-dose albendazole for 28 days and a single dose of ivermectin.

## The Study

The reference Ethics Committee (Comitato Etico per la Sperimentazione Clinica delle Province di Verona e Rovigo) approved this study in July 2016 (study protocol no. 33908). We reviewed the medical records of patients with loiasis admitted to IRCCS Sacro Cuore Don Calabria Hospital (Negrar di Valpolicella, Italy) during 1993–2016 who had been treated with albendazole (400 mg 2×/d for 28 days), followed by ivermectin, (200 µg/kg, in single or multiple doses). For study purposes, we defined a case of loiasis by having previously stayed in an endemic country plus meeting >1 of 3 criteria: 1) eyeworm observed in the eye conjunctiva within the previous 2 months; 2) detection of *L. loa* microfilariae in the peripheral blood, determined by the leukoconcentration method (processing 13 mL of venous blood, with density assessed by examining Giemsa-stained thick smears, prepared with 100 µL of blood); or 3) Calabar swellings associated with eosinophilia (>500 eosinophils/µL) within the previous 2 months. In addition, only patients with >12 months of follow-up were included. 

A total of 23 patients were considered eligible for inclusion. We excluded 7 of them because they had <12 months of follow-up. We included the remaining 16 patients (6 migrants and 10 expatriates) in the analysis (Table). Eleven patients had *L. loa* microfilariae in their blood; 5 had Calabar swellings and eosinophilia; none had an observed eyeworm. Of note, all 6 migrants had eosinophil counts of 500–1,500/µL, whereas all expatriates had eosinophil counts >1,500/µL, in line with previous observations (*10*). 

**Table:** Clinical and laboratory characteristics of 16 patients with imported loiasis treated with albendazole and ivermectin, Italy, 1993–2016*

Pt no.	Age, y/sex	Status	Place of infection	Year of diagnosis	Calabar swelling	EOS/µL	Antifilarial antibodies, OD*	MFF/ mL	Co-infections	Other treatments	Follow-up period
1	64/M	E	CAR	2005	Yes	4,650	5.06	Neg	Giardiasis, strongyloidiasis	Tinidazole	12 y
2	28/F	E	Gabon	2008	Yes	3,300	4.79	Neg	None	None	10 y
3	56/M	E	Chad	2008	No	4,500	4.22	270	Amebiasis	Tinidazole, paromomycin	4 y
4	28/F	M	Angola	2010	No	790	None	263	None	None	14 mo
5	58/M	E	CAR	2010	Yes	9,320	1.74	Neg	None	None	8 y
6	52/M	E	Cameroon	2010	Yes	2,940	5.10	1	None	None	8 y
7	55/M	E	DRC	2011	Yes	2,040	5.21	9	None	None	7 y
8	49/M	E	Congo	2014	Yes	4,930	4.31	Neg	None	None	4 y
9	67/F	E	Nigeria	2014	Yes	13,100	4.05	Neg	Schistosomiasis, strongyloidiasis	Praziquantel	4 y
10	69/M	E	Cameroon	2015	Yes	1,620	2.19	152	None	None	3 y
11†	24/M	M	Nigeria	2015	No	1,300	2.16	160	Hookworm, latent tuberculosis	DEC	26 mo
12‡	27/M	M	Cameroon	2015	No	700	1.53	491	Hookworm, schistosomiasis	Praziquantel	26 mo
13	75/M	E	Cameroon	2015	Yes	4,100	2.85	Neg	None	None	18 mo
14	29/M	M	Cameroon	2016	No	590	2.85	990	Hookworm, schistosomiasis, HMS	Praziquantel, atovaquone/ proguanile	19 mo
15	22/M	M	Cameroon	2016	No	500	1.39	9,800	Schistosomiasis, *Mansonellosis perstans*, chronic HBV	Praziquantel, mebendazole	18 mo
16	18/M	M	Nigeria	2016	No	1,200	2.52	477	Hookworm	None	16 mo

*CAR, Central African Republic; DEC, diethylcarbamazine; DRC, Democratic Republic of the Congo; E, expatriate; EOS, eosinophils; HBV, hepatitis B virus; HMS, hyperreactive malarial splenomegaly; M, migrant; MFF, microfilariae; Neg, negative; OD, optical density; Pos, positive: Pt, patient.  *OD test is positive at ≥1 s. †Patient with failure of albendazole and ivermectin treatment. ‡Received 1 additional course of ivermectin.

A commercial ELISA test, based on *Acanthocheilonema vitae* as source of antigens (Bordier Affinity Products, http://www.bordier.ch), returned positive results for all patients. We also evaluated serologic results retrospectively for patients diagnosed before 2014. Liver function (alanine transaminase and aspartate transaminase levels) had been checked after ≈2 weeks from the onset of treatment with albendazole; no changes were observed in any patient. We recommended that blood tests be repeated every 2 months until patients were free of microfilaremia and that patients make annual follow-up visits for 4–5 years. However, these recommendations were seldom followed, and follow-up visits had to be tailored to each patient’s characteristics and needs. One patient received a further course of ivermectin due to the persistence of low-level microfilaremia 4 months after treatment; testing at subsequent follow-up visits showed no microfilaremia. No severe adverse events were reported, although 2 patients reported itching. 

Posttreatment follow-up periods ranged from 14 months to 12 years. Within 6 months after the end of the treatment, 15 (93.8%) of 16 patients had recovered completely, as demonstrated by disappearance of symptoms, normalization of eosinophil counts, and negative microfilaremia. The remaining patient reported presence of Calabar swelling and microfilaremia 1 month after treatment, so he received a single course of diethylcarbamazine, after which symptoms disappeared within 2 months. 

## Conclusions

Our retrospective study indicates that using the combination of albendazole and ivermectin resulted in a high rate of recovery (15/16, 93.8%) in patients with loiasis with microfilaremia <20,000/mL. By comparison, a study by Klion et al. found that only 38% of 32 patients treated with diethylcarbamazine were cured after 1 course of therapy, and some patients continued to be symptomatic despite >4 courses of treatment (*5*). Three of these symptomatic patients, unresponsive to diethylcarbamazine, were administered albendazole (200 mg 2×/d for 21 d) and then recovered (*8*). Moreover, Klion et al. performed a double-blind, placebo-controlled trial to assess the filaricidal activity and clinical safety of albendazole at the 200 mg dosage (*11*). Within 6 months, albendazole treatment reduced microfilaremia substantially, although not completely. After 6 months, microfilarial density was at 20% of pretreatment level in the albendazole treatment group compared with 84.8% in the placebo group. Ivermectin has been administered alone only to substantially reduce microfilaremia levels, not as a cure, because macrofilaricidal action was not demonstrated (*12*,*13*). It was on the basis of these earlier reports, and because of the unavailability of diethylcarbamazine in Italy, that in 2004 we decided to treat our loiasis patients with a regimen of albendazole at a higher dosage (400 mg 2×/d) for a longer period (28 d), followed by a single dose of ivermectin (200 µg/kg) (*14*).

The main limitations of this study are the retrospective design and the low number of patients included. Moreover, only 1 patient had a microfilaremia level of ≈10,000/mL; however, this level was still lower than the threshold of 20,000/mL established by Kamgno et al. to avoid severe adverse events (*15*). Hence, in our patients, ivermectin could have been administered safely before albendazole. However, we cannot recommend this treatment schedule for patients with microfilaremia >20,000/mL. 

Because data are scant, additional information about the treatment and outcome of loiasis imported into nonendemic countries is useful for clinicians. A previous study pointed out wide heterogeneity of treatment strategies for imported loiasis (*16*), highlighting the need for guidelines for treating loiasis in nonendemic areas, where there is no risk of reinfection. 

In conclusion, a combination of high-dose albendazole (400 mg 2×/d for 28 d) plus a single 200 µg/kg dose of ivermectin might be recommended for loiasis patients with microfilaremia <20,000/mL as an alternative to diethylcarbamazine, which is the treatment of choice for loiasis but is often unavailable. In case of clinical cure (absence of symptoms) but persistence of low levels of microfilaremia, an option might be a second course of ivermectin to achieve clearance of microfilariae (*9*). 
